# Bioactive Phenolic Amides from *Celtisafricana*

**DOI:** 10.3390/molecules17032675

**Published:** 2012-03-05

**Authors:** Areej Mohammad Al-Taweel, Shagufta Perveen, Azza Muhammed El-Shafae, Ghada Ahmed Fawzy, Abdul Malik, Nighat Afza, Lubna Iqbal, Mehreen Latif

**Affiliations:** 1 Department of Pharmacogonosy, College of Pharmacy, King Saud University, Riyadh, P.O. Box 2457, Riyadh 11451, Saudi Arabia; 2 Department of Pharmacognosy, Faculty of Pharmacy, Cairo University, Cairo 11562, Egypt; 3 International Centre for Chemical Sciences, H.E.J. Research Institute of Chemistry, University of Karachi, Karachi 75270, Pakistan; 4 Department of Chemistry, University of Karachi, Karachi 75270, Pakistan

**Keywords:** *Celtis africana*, antioxidant, anti-inflammatory, acetylcholinestrease enzyme inhibition

## Abstract

Nine compounds have been isolated for the first time from *Celtis africana*, namely *trans*-*N*-coumaroyltyramine (**1**), *trans*-*N*-feruloyltyramine (**2**), *trans*-*N*-caffeoyltyramine (**3**), lauric acid (**4**), oleic acid (**5**), palmitic acid (**6**), lupeol (**7**), *β*-sitosterol (**8**) and oleanolic acid (**9**), respectively. Their structures have been elucidated by different spectroscopic techniques. The isolated compounds were screened for their antioxidant, anti-inflammatory and acetylcholinestrease enzyme inhibitory activities. Compounds **1**–**3** showed significant antioxidant and anti-inflammatory activities and weak to moderate acetylcholinestrease enzyme inhibition activity.

## 1. Introduction

**Figure 1:**
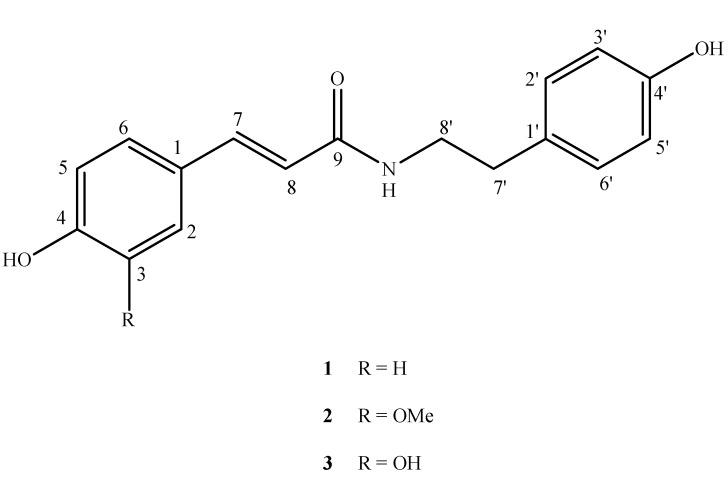
Structures of compounds **1**–**3**.

*Celtis africana* Burm. f. is common and widespread in South Africa where the leaves are used as a traditional human and veterinary medicine for the treatment of indigestion and edema [1]. The sun-dried bark and roots are powdered and infused in water or milk and taken orally every day by the patients for the treatment of cancer in South Africa [2]. A literature survey revealed that very little chemical work has been carried out on this plant, and only *C*-glycosylflavonoids have been isolated from *the* *n*-butanol soluble fraction of *C. africana*. [3]. The methanol extracts of the leaves and stems of *C. africana* showed strong DPPH radical scavenging activity, but no work has been done on the activity of pure isolates [4]. In the biological screening of different fractions of the ethanol extract, the chloroform soluble sub-fraction showed anti-inflammatory, antioxidant and acetylcholinestrease enzyme inhibiton activities. This prompted us to carry out bioassay-directed isolation studies on the chloroform soluble fraction of *C. africana*. As a result, nine compounds have been isolated for the first time from *C. africana*, namely *trans*-*N*-coumaroyltyramine (**1**), [5] *trans*-*N*-feruloyltyramine (**2**), [6] *trans*-*N*-caffeoyltyramine (**3**), [7] lauric acid (**4**), oleic acid (**5**) [8], palmitic acid (**6**) [9], lupeol (**7**), *β*-sitosterol (**8**) [10] and oleanolic acid (**9**) [11], respectively (Figure 1). The isolated compounds were screened for their antioxidant, anti-inflammatory and acetylcholinestrease enzyme inhibitory activities.

## 2. Results and Discussion

### 2.1. Structure Elucidation and Biological Assay of Compounds ***1***–***3***

The chloroform soluble fraction of *C. africana* was subjected to column chromatography over silica gel eluting with different mobile phases. Compounds **1**–**9** were finally obtained and their structures were established by mass and NMR spectroscopy including 2D NMR techniques.

Compound **1** was isolated as a white amorphous solid and the molecular formula C_17_H_17_NO_3_ was established by HREIMS showing M^+^ peak at *m/z* 283.1184 (calcd. for C_17_H_17_NO_3_, 283.1208). The ^1^H-NMR spectrum of **1** showed resonances for two sets of AA′BB′ type signals at *δ* 7.41 (2H, d, *J* = 8.4 Hz, H-2, 6), 7.06 (2H, d, *J* = 8.6 Hz, H-2′, 6′), 6.80 (2H, d, *J* = 8.4 Hz, H-3, 5) and 6.73 (2H, d, *J* = 8.6 Hz, H-3', 5'). In addition, two coupled triplets of methylene protons appeared at *δ* 2.76 and 3.46 (each 2H, t, *J* = 7.5 Hz) for H-7 and H-8, respectively. The ^1^H-NMR spectrum of **1** further showed olefinic protons at *δ* 7.44 and 6.38 (each, 1H, d, *J* = 15.5 Hz), the larger coupling constant indicated *trans* configuration of the double bond. Therefore, compound **1** was identified as *trans*-*N*-coumaroyltyramine by its NMR data and comparison with the published data in the literature [5].

Compound **2** showed similar NMR data with those of compound **1**, except for one ABX type signal pattern in the ^1^H-NMR spectrum which appeared at *δ* 6.81 (1H, d, *J* = 8.5 Hz, H-5), 7.13 (1H, d, *J* = 1.2 Hz, H-2) and 7.04 (1H, dd, *J* = 8.5, 1.2 Hz, H-6) due to the presence of an additional methoxy group at δ 3.85 (3H, s, OCH3). Compound **2** was identified as *trans*-*N*-feruloyltyramine by its ^13^C-NMR data, which showed complete agreement to those reported in literature [6].

Compound **3** was obtained as white crystals and gave a peak at 299 in EIMS. A comparison of the ^1^H- and ^13^C-NMR spectral data with those of **2** and **3** revealed the replacement of methoxyl group by a hydroxyl group at position-3. Based on these analysis and comparison of the data with the literature [7], compound **3** was identified as *trans*-*N*-caffeoyltyramine.

The antioxidant activity of compounds **1**–**3** was measured by the 1,1-diphenyl-2-picrylhydrazyl (DPPH) method. Compounds **2** and **3** reflected significant antioxidant ability when compared to BHA used as standard. The data shows that the numbers of hydroxyl group are apparently the controlling factors for antioxidant activity. Since the compound **3** has *ortho* hydroxyl on the phenyl ring then it show higher antioxidant activity compared to the others (Table 1).

**Table 1:** *IC_50_* (µM) values of compounds **1**–**3** in antioxidant assay.

Compounds	DPPH Scavenging Activity IC_50_^a^ [μM]
****1****	62.0 ± 0.15
****2****	33.2 ± 0.14
****3****	26.3 ± 0.32
****BHA**** ^b^	44.3 ± 0.09

^a^ Values ± SEM (standard mean error of 3 assays); ^b^ Standard DPPH scavenging activity.

The anti-inflammatory activity of **1**–**3** was determined in carrageen induced paw edema of rats and found to be significant compared to diclofenac sodium used as control (Table 2).

**Table 2:** Anti-inflammatory potential of compounds **1**–**3** in carrageenan induced paw edema of rats.

Group (3 rats in each)	Treatment10 mg/kg	Edema Volume(V_c_ = V_f_ − V_0_)	Percent Inhibition(%)
1	Cage-1 control	0	
2	Diclofenic Sodium	0.22 ± 0.05	57.6
3	**1**	0.26 ± 0.24	48.3
4	**2**	0.35 ± 0.22	32.6
5	**3**	0.28 ± 0.11	25.5

According to the obtained results, the examined amides **1**–**3** are weak to moderate acetylcholinesterase inhibitors, with the *IC_50_* values 98.3, 86.0 and 84.3 μM (Table 3). Comparing the acetylcholinesterase inhibitory activity of compounds **1**–**3**, it is found that compound **1** with the hydroxyl group at position 4 has moderate activity and it is weaker than **2**, which has an additional methoxy group at position 3. On the contrary, compound **3** with two hydroxy groups at position 3 and 4 showed stronger activity compared with its methoxy derivative **2**. These results showed that the additional hydroxyl group at C-3 increases the inhibitory activity of compound **3**. Compound **1** was previously isolated from the *Celtis chinensis* and inhibited AChE activity in a dose-dependent manner with an IC_50_ value of 34.5 mg/mL [12].

**Table 3:** *In vitro* inhibition of AChE by compounds **1**–**3**.

Compounds	AChE IC_50_^a^ [μM]
****1****	98.3 ± 0.21
****2****	86.0 ± 0.34
****3****	84.3 ± 0.32
****Galanthamine****	32.3 ± 2.3

^a^ IC_50_ values are the mean ± standard error mean (S.E.M.) of three assays.

## 3. Experimental

### 3.1. General

The ^1^H-, ^13^C-NMR, HMQC, and HMBC spectra were recorded on Bruker spectrometers (AMX) operating at 500 MHz for ^1^H-NMR and 125 MHz for ^13^C-NMR, respectively. The chemical shift values are reported in ppm (δ) units and the coupling constants (*J*) are in Hz. EIMS and HREIMS were recorded on JMS-HX-110 and JMS-DA 5000 mass spectrometers. Aluminum sheets precoated with silica gel 60 F_254_ (20 × 20 cm, 0.2 mm thick; E-Merck) were used for TLC and silica gel (230–400 mesh) was used for column chromatography. Visualization of the TLC plates was carried out under UV at 254 and 366 nm and by spraying with ceric sulfate reagent solution (with heating).

### 3.2. Plant Material

The aerial parts of *C. africana* (2.5 kg, dry weight) were collected (March 2009) from Riyadh (Saudi Arabia) and air-dried. The identity of the plant was verified by M. Atiqur El-Rahman, Plant Taxonomist, College of Pharmacy, King Saud University. A voucher specimen (No. 44) was deposited in the herbarium of Department of Pharmacogonsy, King Saud University.

### 3.3. Extraction and Isolation

The aerial parts of *C. africana* (2.5 kg) were shade-dried, ground and extracted at room temperature with ethanol-water (8:2, thrice). The combined ethanol extract (100 g) was divided into *n*-hexane (30 g), chloroform (20 g), *n*-butanol (30 g) and water (20 g) soluble sub-fractions. A part of CHCl_3_ soluble sub-fraction (17 g) was loaded on a silica gel column and the elution was successively carried out with mixture of *n*-hexane-chloroform, chloroform and mixture of chloroform-methanol in increasing order of polarity leading to eight major sub-fractions I–VIII. Fraction I, which eluted with *n*-hexane-chloroform (8:2) showed three major spots on TLC. It was further subjected to column chromatography using *n*-hexane-chloroform (7:3) as eluent to afford compound **4** (15 mg), compound **5** (14 mg) and compound **6** (20 mg). The fraction II obtained from *n*-hexane-chloroform (6:4) was further purified by column chromatography eluting with *n*-hexane-chloroform (5.5:4.5) to afford compounds **7** (22 mg) and **8** (25 mg). The fraction III obtained from *n*-hexane-chloroform (1:1) was further purified by column chromatography eluting with *n*-hexane-chloroform (4:6) to afford compound **9** (22 mg). The fraction IV obtained from chloroform was a mixture of two components, which were separated by column chromatography using solvent system chloroform-methanol (9.8:0.2) to afford compounds **1** (30 mg) and **2** (25 mg) from the top and the tail fractions, respectively. Compound **3** (26 mg) was obtained from fraction V by column chromatography over silica gel using chloroform-methanol (9.5:0.5).

### 3.4. Biological Bioassay

#### 3.4.1. Antioxidant Assay

The free radical scavenging activity was measured by the 1,1-diphenyl-2-picrylhydrazyl (DPPH) assay [13]. A 0.3 μM solution of DPPH in ethanol was prepared. Each sample (5 µL) of different concentration (62.5–500 μg) was mixed with DPPH solution (95 µL). The mixture was dispersed in 96 well plates and incubated at 37 °C for 30 min. The absorbance at 515 nm was measured by micro liter plate reader (Spectramax plus 384 Molecular Devices, Sunnyvale, CA, USA) and percent radical scavenging activity was determined in comparison with the methanol treated control. BHA is used as standard.

DPPH scavenging effect (%) = Ac − As/Ac × 100

where Ac = Absorbance of control (DMSO treated); As = Absorbance of sample.

#### 3.4.2. *In Vivo* Anti-Inflammatory Assay

The anti-inflammatory potential of compounds **1**–**3** was evaluated in rats by using the following chemicals: carrageenan and dimethyl sulfoxide (DMSO) obtained from Sigma and diclofenac sodium (Novartis Pharmaceuticals). Wistar rats (120–180 g) of either sex were used obtained from the animal house of Agha Khan University of Karachi. Animals were kept under standard environmental conditions with free access to food and water. Rats were divided into five groups (six in each group). Group 1 served as control and received 0.9% saline while group 2 received diclofenac sodium orally as a standard drug. Groups 3, 4 and 5 were administered compounds **1**–**3**, 10 mg/kg orally. Acute edema was induced in the right hind paw of rats by injecting 0.1 mL of freshly prepared carrageenan 1% solution subcutaneously in the planter region. Drugs were given 1 h before carrageenan challenge. Paw volume was measured using a piethysmometer at 0 and 4 h after carrageenan injection. The edema volume and percent inhibition in edema was calculated as indicated by Christopher [14]. 

#### 3.4.3.*In Vitro* Acetylcholinesterase Inhibition Assay

Acetylcholinesterase (electric-eel EC 3.1.1.7), acetylthiocholine iodide, 5,5'-dithiobis[2-nitrobenzoic-acid] (DTNB) and galanthamine were purchased from Sigma (St. Louis, MO, USA). Buffers and other chemicals were of analytical grade. Acetylcholinesterase inhibiting activity was measured according to a slightly modified spectrophotometric method. Acetylthiocholine iodide was used as substrates in the assay. 5,5'-Dithiobis[2-nitrobenzoic-acid] (DTNB) was used for the measurement of cholinesterase activity. 0.1 mM sodium phosphate buffer (pH 8.0, 140 μL), 3 mM DTNB (10 μL), test compound solution (20 μL, 1 mg/mL) and 15 mM acetylcholinesterase solution (20 μL) was mixed and incubated for 15 minutes (25 °C). The reaction was then initiated by the addition of acetylthiocholine (10 μL). The hydrolysis of acetylthiocholine was monitored at 412 nm after 20 min. All the reactions were performed in triplicate in 96-well microliter plates in SpectraMax 340 (Molecular Devices) [15].

#### 3.4.4. Estimation of *IC_50_* Values

The concentrations of test compounds that inhibited the hydrolysis of substrate (acetylthiocholine) by 50% (*IC_50_*) were determined by monitoring the effect of increasing concentrations of these compounds in assays of the inhibition values. The percentage inhibition was calculated using the equation:

Inhibition (%) = 1 − (A_sample_/A_control_) × 100

where A_sample_ is the absorbance of the sample and A_control_ is the absorbance of the blank (methanol in phosphate Buffer, pH 8). The *IC_50_* values were then calculated using the EZ-Fit Enzyme Kinetics program (Perrella Scientific Inc., Amherst, MA, USA).

### 3.5. Spectral Data

*trans-N-Coumaroyltyramine* (**1**). White solid (30 mg); m.p. 255–256 °C; EIMS (*m/z*): 283 [M^+^] (16), 176, 164, 147 (100); ^1^H-NMR (CD_3_OD): *δ* 7.06 (d, 2H, *J* = 8.6 Hz, H-2', 6'), 7.41 (2H, d, *J* = 8.4 Hz, H-2, 6), 6.73 (2H, d, *J* = 8.6 Hz, H-3', 5'), 6.80 (2H, d, *J* = 8.4 Hz, H-3, 5), 7.44 (1H, d, *J* = 15.5 Hz, H-8); 6.38 (d, 1H, *J* = 15.5 Hz, H-7), 3.46 (t, 2H, *J* = 7.5 Hz, H-8'), 2.75 (t, 2H, *J* = 7.5 Hz, H-7'); ^13^C-NMR (CD_3_OD): *δ* 169.2 (C-9), 160.5 (C-4), 156.9 (C-4'), 141.8 (C-7), 130.7 (C-2', 6'), 131.3 (C-1'), 130.5 (C-2, 6), 127.7 (C-1), 118.4 (C-8), 116.7 (C-3', 5'), 116.2 (C-3, 5), 42.5 (C-8'), 35.8 (C-7'). 

*trans-N-Feruloyltyramine* (**2**). White solid (25 mg); m.p. 145–147 °C; EIMS *m/z* (rel. int.): 313 [M]^+^(15), 177 (100), 145 (22), 120 (18), 107 (19); ^1^H-NMR (CD_3_OD): *δ* 7.44 (d, 1H, *J* = 15.5 Hz, H-7), 7.13 (d, 1H, *J* = 1.2 Hz, H-2), 7.07 (d, 2H, *J* = 8.4 Hz, H-2', 6'), 7.048 (dd, 1H, *J* = 8.5, 1.2 Hz, H-6), 6.81 (d, 1H, *J* = 8.5 Hz, H-5), 6.73 (d, 2H, *J* = 8.4 Hz, H-3', 5'), 6.41 (d, 1H, *J* = 15.5 Hz, H-8), 3.85 (3H, s, OCH3), 3.47 (t, 2H, *J* = 7.5 Hz, H-8), 2.76 (t, 2H, *J* = 7.5 Hz, H-7), ^13^C-NMR (CD_3_OD): *δ* 169.2 (C-9), 156.9 (C-4'), 149.8 (C-4), 149.3 (C-3), 142.0 (C-7), 131.3 (C-1'), 130.7 (C-2', 6'), 128.2 (C-1), 123.2 (C-6), 118.7 (C-8), 116.4 (C-5), 116.2 (C-3', 5'), 111.5 (C-2), 56.4 (OCH3), 42.5 (C-8'), 35.8 (C-7'). 

*trans-N-Caffeoyltyramine* (**3**). White solid (26 mg); m.p. 215–217 °C; EIMS *m/z* (rel. int.): 299 [M]^+^ (25), 180 (48), 163 (100). 120 (17); ^1^H-NMR (CD_3_OD): *δ* 7.41 (d, 1H, *J* = 15.6 Hz, H-7), 7.06 (d, 1H, *J* = 1.0 Hz, H-2), 7.02 (d, 2H, *J* = 8.5 Hz, H-2', 6'), 6.98 (dd, 1H, *J* = 8.4, 1.0 Hz, H-6), 6.79 (d, 1H, *J* = 8.4 Hz, H-5), 6.75 (d, 2H, *J* = 8.5 Hz, H-3', 5'), 6.40 (d, 1H, *J* = 15.6 Hz, H-8), 3.47 (t, 2H, *J* = 7.5 Hz, H-8), 2.76 (t, 2H, *J* = 7.5 Hz, H-7), ^13^C-NMR (CD_3_OD): *δ* 169.3 (C-9), 156.7 (C-4'), 148.5 (C-4), 147.7 (C-3), 141.6 (C-7), 131.0 (C-1′), 130.7 (C-2', 6'), 127.1 (C-1), 122.1 (C-6), 118.5 (C-8), 116.3 (C-5), 116.4 (C-3', 5'), 114.5 (C-2), 42.4 (C-8'), 35.7 (C-7').

## 4. Conclusions

In summary, we have evaluated the anti-inflammatory, antioxidant and acetylcholinestrease enzyme inhibitory activities of three phenolic amides isolated for the first time from *C. africana*. These compounds showed potent anti-inflammatory and antioxidant potential, and moderate acetylcholinestrease enzyme inhibition activity.
